# The Reliability and Stability of an Inferred Phylogenetic Tree from Empirical Data

**DOI:** 10.1093/molbev/msw272

**Published:** 2017-01-18

**Authors:** Yukako Katsura, Craig E. Stanley, Sudhir Kumar, Masatoshi Nei

**Affiliations:** 1 Department of Biology and Institute for Genomics and Evolutionary Medicine, Temple University, Philadelphia, PA; 2 Department of Biology and Institute of Molecular Evolutionary Genetics, Pennsylvania State University, State College, PA

**Keywords:** phylogenetic trees, reliability, stability, subtrees, bootstrap probability, MHC class II β chain genes, computer program RESTA.

## Abstract

The reliability of a phylogenetic tree obtained from empirical data is usually measured by the bootstrap probability (Pb) of interior branches of the tree. If the bootstrap probability is high for most branches, the tree is considered to be reliable. If some interior branches show relatively low bootstrap probabilities, we are not sure that the inferred tree is really reliable. Here, we propose another quantity measuring the reliability of the tree called the stability of a subtree. This quantity refers to the probability of obtaining a subtree (Ps) of an inferred tree obtained. We then show that if the tree is to be reliable, both Pb and Ps must be high. We also show that Ps is given by a bootstrap probability of the subtree with the closest outgroup sequence, and computer program RESTA for computing the Pb and Ps values will be presented.

## Introduction

**Fig. 1:**
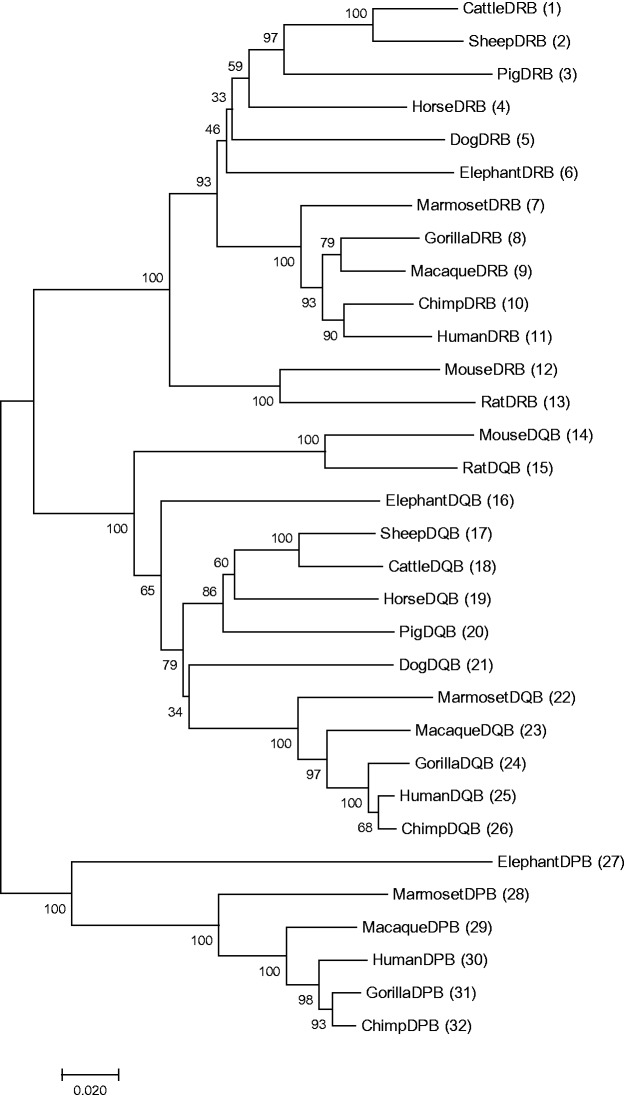
Phylogenetic tree of MHC class II DRB, DQB, and DPB genes in mammals. The tree and Pb values were computed by the NJ method with p distance (NJp method) using 32 DNA sequences for 13 mammalian species. The number of nucleotide sites used was 1,320 bp per sequence. The Pb value is given for each interior branch, and the number of bootstrap replications for a subtree was 1,000. The aligned nucleotide sequences are available as example data in RESTA.

The purpose of this article is to examine the reliability of an inferred tree from empirical data. To make our question concrete, let us consider the phylogenetic tree representing the evolution of major histocompatibility complex (MHC) class II β chain genes in mammals (see fig. 1). The MHC genes are immune system genes and present foreign peptides to T-cell cytotoxic lymphocytes, thereby triggering appropriate immune responses. MHC genes can be classified into class I and class II genes, and the class II genes can further be divided into the DP, DM, DO, DQ, and DR region genes in mammals (Kulski et al. 2002; Shiina et al. 2009). Furthermore, each of these DNA regions contains the α and β chain genes (Klein and Figueroa 1986; Nei and Hughes 1991). Here, we consider a phylogenetic tree of only class II β chain genes.

Figure 1 shows the phylogenetic tree obtained by the NJp method (Saitou and Nei 1987; Yoshida and Nei 2016) for the three major groups of MHC class II β chain genes (DPB, DQB, and DRB) in mammals (Hughes and Nei 1990; Takahashi et al. 2000). The human genome is known to have four DRB, three DQB, and two DPB genes (Shiina et al. 2009), but here we use only DRB1, DQB1, and DPB1 genes. DPB genes are nonfunctional (psudogenes) in rodents and carnivores (Yuhki et al. 2003; Debenham et al. 2005), and absent from the currently known mammalian genomes except in primates and elephant (Wilming et al. 2013). They are believed to have lost their function in the process of evolution.

**Fig. 2:**
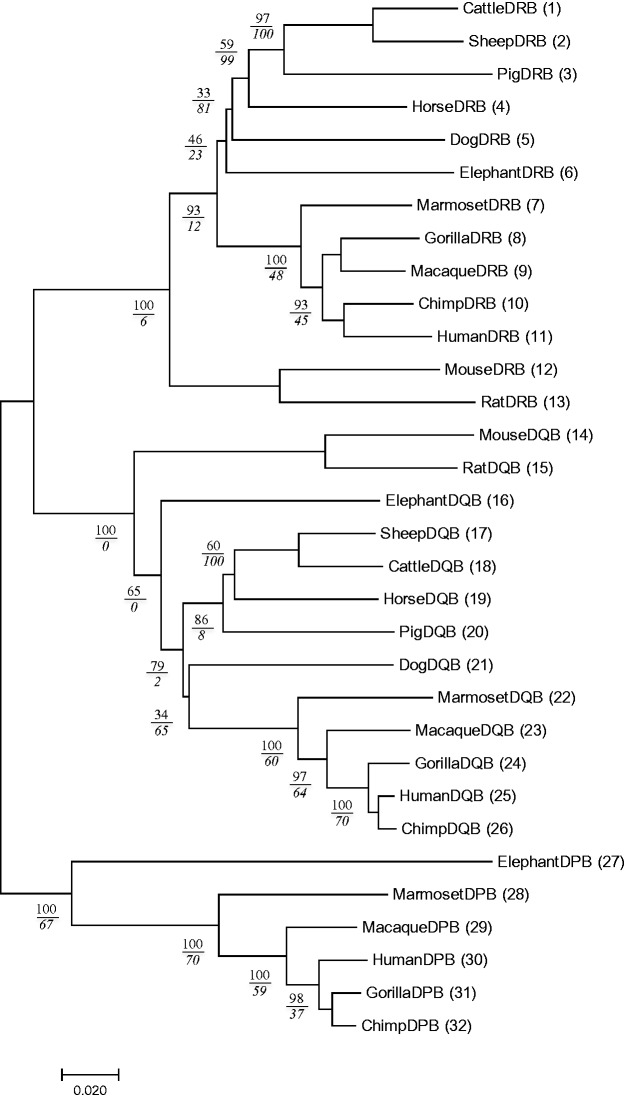
Phylogenetic tree of MHC class II β chain genes in mammals with the Pb and Ps values. The Ps value is given as an italic number below the Pb value for each relevant interior branch. The Pb values are the same as those in figure 1.

In figure 1, the number given for each interior branch indicates the usual bootstrap probability (Pb) of the branch when the entire set of sequences is used (Felsenstein 1985). Some interior branches have high Pb values, whereas the others do not. In this case, we are not sure whether the tree is reliable or not. In some cases, even if Pb is high, some parts of the tree may not be so reliable as we wish because the Pb value merely represents the probability of partitioning of the entire sequences at the relevant interior branch (Nei and Kumar 2000). For example, the interior branch for the subtree of the cattle, sheep, and pig DRB1 genes (sequences 1, 2, and 3) has a value of Pb = 97%, and the subtree for cattle and sheep genes (sequences 1 and 2) has a bootstrap probability of Pb = 100%. These values suggest that the cluster or the subtree of sequences 1, 2, and 3 is highly reliable. Let us now test this hypothesis by using sequence 4 as the closest outgroup. A simple way of testing this hypothesis is to conduct a bootstrap test of the subtree using the closest outgroup gene of the subtree (Ps). In the present case, we have used 1,000 replications for the bootstrap test, and Ps is expressed as a percentage. (In the present case, we recommend that 500 or more replications be used to obtain an accurate Ps value.) The result of our test is presented in figure 2, the Ps value being 100%. This Ps value supports our hypothesis, and the subtree of sequences 1, 2, and 3 is highly reliable. In this article, the probability of obtaining the same topology as that of the original subtree will be called the stability (Ps) of the subtree and expressed as a percentage.

The purpose of this article is to compute the Pb and Ps values for all relevant interior branches and examine their values and relationships. The computer program RESTA for computing the Pb and Ps values will also be presented.

## Results and Discussion

Although we have shown how to compute the Ps value for one case, let us first continue this process for the next few steps. The Ps value of the subtree of sequences 1–4 is computed by using sequence 5 as the closest outgroup. However, the actual computation of Ps is done first by making an unrooted tree of sequences 1–5. Let us designate the total number of replications producing this subtree by N. We now consider the number of replications in which the topology of the subtree becomes identical with that of the original subtree and designate the number of the replications by s. The Ps value for the subtree is then given by Ps = (s/N) × 100%. In the present case, it becomes 99%. Figure 2 shows the Ps value listed as the italic and lower number of the paired values for the appropriate interior branch, the upper number referring to the Pb value. In the present case, Ps is 99% and is higher than Pb (=59%). Next, we compute the Ps value of the subtree of sequences 1–5 by using sequence 6 as the closest outgroup, and we obtain a value of 81%. This value is again higher than the Pb value (=33%). If we conduct a bootstrap test for each subtree, we obtain Ps values for all relevant interior branches, as listed in figure 2. In the case of the subtree of sequences 1–11, Ps is 12% and is much lower than Pb (= 93%). This has happened apparently because it is difficult to maintain the same subtree structure when a large number of sequences are involved in the subtree.

In the computation of Ps, it is possible that two or more outgroup sequences exist. For example, in the computation of the Ps value for the subtree of sequences 1–6, one may use any of the sequences 7, 8, 9, 10, and 11 as an outgroup. In the present article, we used one of the five possible outgroup sequences at a time and computed the Ps values. We then took the average of the five Ps values. Actually, we noticed that the Ps value varies considerably with outgroup sequence, and the average Ps value looked to be better than the Ps value for a randomly chosen gene as the outgroup.

Figure 2 shows that Pb is higher than Ps for some interior branches, but it is not so for others. This indicates that the accuracy of a subtree is not so high as suggested by Pb, and in some cases, the accuracy of a subtree is very low. Only when both Pb and Ps are high, can we trust the subtree structure. We should know that a subtree with a 100% of Pb value can have a 0% of the Ps value. This indicates the importance of computing the Ps value. In figure 2, we have not computed the Ps value when there are only two sequences in the subtree because a subtree of two sequences always produces the same tree.

**Fig. 3:**
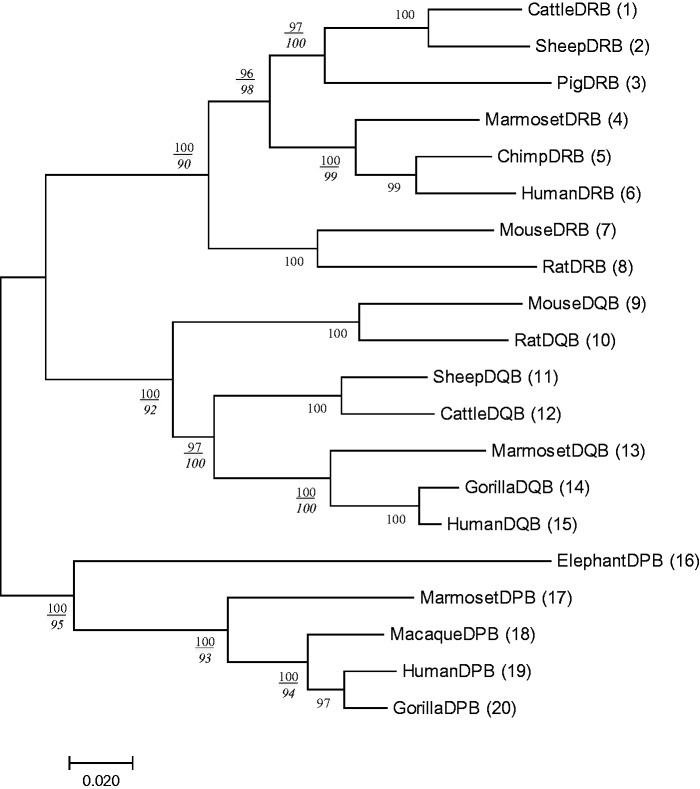
Phylogenetic tree of MHC class II β chain genes in mammals with a high Pb value (>97%) for all interior branches. Twenty sequences were selected from those of Figure 1. The Ps value is shown as an italic number below the Pb value for each relevant interior branch.

Previously, we stated that if most interior branches show a high Pb values, the tree would be reliable. Let us examine the validity of this statement. For this purpose, we constructed a tree with Pb > 0.97 for all interior blanches by deleting some of the sequences used in figure 1. A resulting tree with 20 sequences is presented in figure 3. Generally speaking, Ps is also quite high in this tree, but it can be smaller than Pb. We can therefore conclude that the computation of Ps is necessary in this case as the computation of Pb is.

**Fig. 4:**
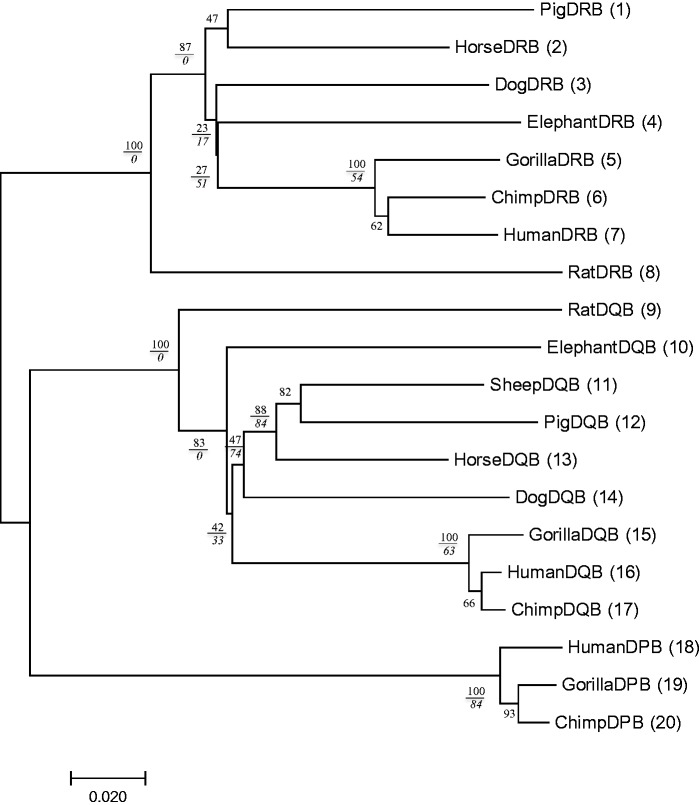
Phylogenetic tree of MHC class II β chain genes in mammals with small Pb values for most interior branches. Twenty sequences were selected from the sequences of Figure 1. The Ps value is shown as an italic number below the Pb value for each relevant interior branch. The Pb value is mostly less than 75%.

Figure 4 represents the opposite case, where Pb is low for most interior branches of the tree. This tree was produced again by deleting some sequences from those of figure 1. However, we should mention that it was difficult to produce a tree with Pb < 75% for all interior branches so that some interior branches have remained to have high Pb values. At any rate, our conclusion is that the Ps values are generally low when Pb is low. In other words, when Pb is small for most interior branches, the tree is not reliable.

In this article, we used the NJp method of tree construction (Saitou and Nei 1987; Yoshida and Nei 2016) for computing the Pb and Ps values, but these values are computable for any tree making method, whether the tree is constructed by the NJp, likelihood, or Bayesian method. However, once a tree is constructed by a particular method, the Pb and Ps values must be computed by the same method.

**Fig. 5:**
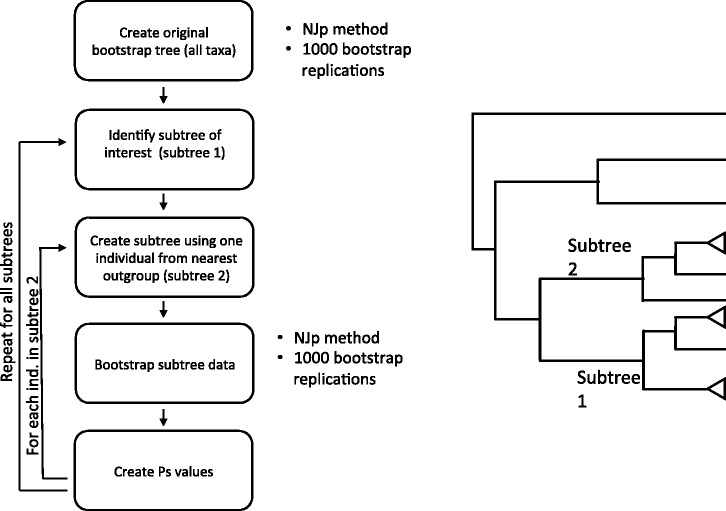
The flowchart of computer program RESTA for computing Pb and Ps values. The computer program RESTA has been produced by following the steps of the flowchart and computes the Pb and Ps values. It is available for download from igem.temple.edu/labs/nei/program/resta (last accessed November 30, 2016) URL and is meant for Linux operating system.

Although the Ps value is computable by the above method, the actual computation is cumbersome and errors can occur when the number of sequences is large. We have therefore developed a computer program for computing Pb and Ps values. This program is called RESTA, and its flowchart is given in figure 5. The computation of Pb and Ps with RESTA will give the same values as those in figures 2–4. The program RESTA can be downloaded from igem.temple.edu/labs/nei/program/resta (last accessed November 30, 2016).
